# GTP-binding of ARL-3 is activated by ARL-13 as a GEF and stabilized by UNC-119

**DOI:** 10.1038/srep24534

**Published:** 2016-04-22

**Authors:** Qing Zhang, Yan Li, Yuxia Zhang, Vicente E. Torres, Peter C. Harris, Kun Ling, Jinghua Hu

**Affiliations:** 1 Department of Nephrology and Hypertension, Mayo Clinic, Rochester, Minnesota, USA; 2 Department of Biochemistry and Molecular Biology, Mayo Clinic, Rochester, Minnesota, USA; 3 Mayo Translational PKD Center Mayo Clinic, Rochester, Minnesota, USA.

## Abstract

Primary cilia are sensory organelles indispensable for organogenesis and tissue pattern formation. Ciliopathy small GTPase ARLs are proposed as prominent ciliary switches, which when disrupted result in dysfunctional cilia, yet how ARLs are activated remain elusive. Here, we discover a novel small GTPase functional module, which contains ARL-3, ARL-13, and UNC-119, localizes near the poorly understood inversin (InV)-like compartment in *C. elegans*. ARL-13 acts synergistically with UNC-119, but antagonistically with ARL-3, in regulating ciliogenesis. We demonstrate that ARL-3 is a unique small GTPase with unusual high intrinsic GDP release but low intrinsic GTP binding rate. Importantly, ARL-13 acts as a nucleotide exchange factor (GEF) of ARL-3, while UNC-119 can stabilize the GTP binding of ARL-3. We further show that excess inactivated ARL-3 compromises ciliogenesis. The findings reveal a novel mechanism that one ciliopathy GTPase ARL-13, as a GEF, coordinates with UNC-119, which may act as a GTP-binding stabilizing factor, to properly activate another GTPase ARL-3 in cilia, a regulatory process indispensable for ciliogenesis.

Understanding the key regulatory steps governing how cilia form is essential for developing intervention strategies for ciliopathies^1^,^2^,^3^. Small GTPases are key cellular switches correlated with various human disorders. The unique feature that small GTPases can toggle a particular signaling pathway on and off by simply changing between GTP-bound and GDP-bound form makes them highly favorable therapeutic targets. For GTPase regulators, GEFs selectively recognize GDP-bound GTPases, while GTPase-activating proteins (GAPs) and GTPase effectors selectively bind GTP-bound GTPases^4^,^5^. We previously found that small GTPase ARL-3 and ARL-13, belonging to the poorly understood ADP-ribosylation factor^6^-like protein (ARL) family within the Ras superfamily (reviewed in)^7^,^8^, play distinct roles in the context of cilia^9^,^10^. In mammalian cells, ARL3 regulates the allosteric release of lipid-modified ciliary cargo proteins^11^,^12^. *Arl3*^−/−^ mice exhibit typical ciliopathy manifestations^13^. Mutations in ARL13B cause Joubert Syndrome disorder and *Arl13b*^−/−^ mouse shows coupled defects in cilia structure and Sonic hedgehog signaling^14^,^15^. In *C. elegans, arl-13* and *arl-3* act antagonistically to regulate intraflagellar transport (IFT) integrity and ciliogenesis^10^. A recent structure study indicates that ARL13 can promote ARL3 GDP release^16^. However, the underlying molecular mechanisms of these ARL GTPases are not clear.

## UNC-119 exclusively localized to Inv-like compartment in cilia

**Figure 1:**
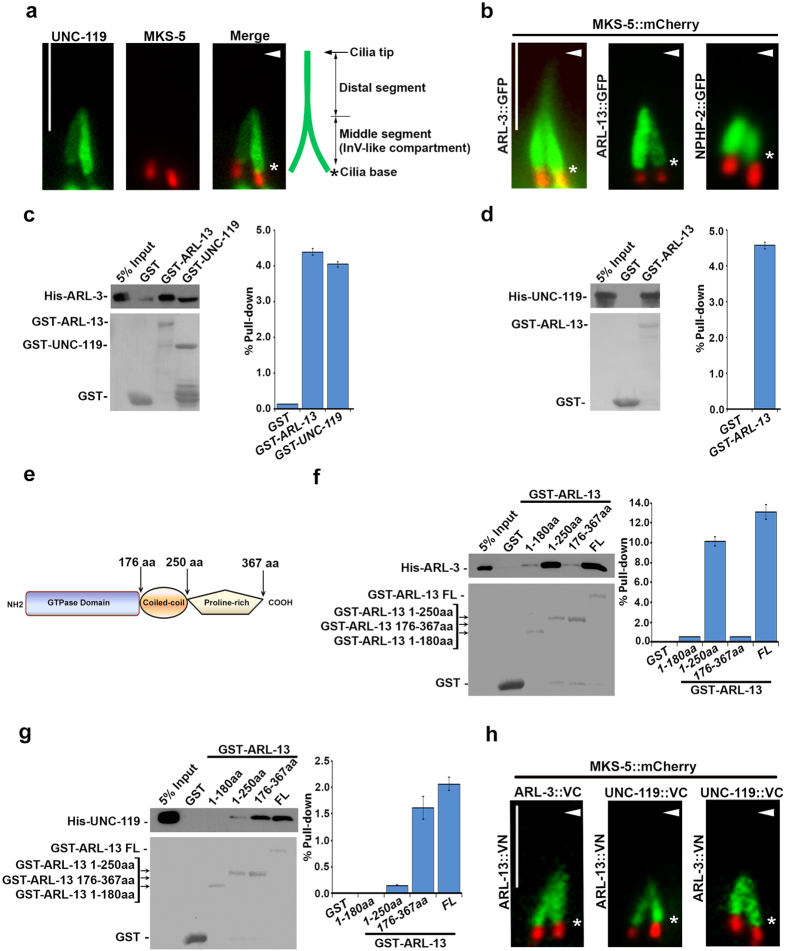
UNC-119, ARL-3 and ARL-13 associate with each other in the InV-like compartment of cilia. (**a**) GFP-tagged UNC-119 specifically localizes to the middle segment of cilia, which is analogous to mammalian inversin compartment. mCherry-tagged MKS-5 was used as a transition zone marker. (**b**) Identical to UNC-119, ARL-13 localizes specifically along the middle segment that is defined by NPHP-2 as the InV-like compartment. ARL-3 localizes along the whole cilium but show more concentrated signal in the InV-like compartment. (**c**,**d**). The direct interactions between UNC-119, ARL-3, and ARL-13 are shown by GST pull-down assays. (**e**) ARL-13 is an atypical GTPase that contains a GTPase domain (1–176 aa), a coiled-coil domain (177–250 aa) and a Proline-rich C-terminus (251–367 aa). (**f**) ARL-13 directly interacts with ARL-3 *via* its N-terminal domain, and both GTPase domain and coiled-coil domain are essential for this association. GST pull down assay was used to determine the binding between His-tagged ARL-3 and GST-fused ARL-13 variants, including GTPase domain of ARL-13 (1–180aa), GTPase and coiled-coil domain of ARL-13 (1–250aa), coiled-coil domain and proline-rich domain of ARL-13 (176–367aa), and full length ARL-13 (FL). (**g**) ARL-13 directly interacts with UNC-119 *via* its C-terminal domain. GST pull down assay was performed to study the interaction between His-tagged UNC-119 and GST-tagged ARL-13 variants as indicated in (**c**). In (**c**,**d**,**f**,**g**), the input and pull-down samples were analyzed with immunoblotting *via* anti-His antibody (upper panel) and Ponceau S staining was used to indicate the GST proteins (lower panel). Quantifications of the pull down efficiency of different proteins were shown in bar graphs. The protein band intensities of individual pull-down sample were compared to the corresponding input sample via Image J to calculate percent of pull-down. (**h**) BiFC analyses in live worms. Strong fluorescence complementation can be visualized in the InV-like compartment of cilia between all BiFC pairs among UNC-119, ARL-3, and ARL-13. VN: Venus N-terminus. VC: Venus C-terminus. All BiFC tags were fused to the carboxy-terminus of proteins. Scale bar: 5 μm. Arrowheads indicate the ciliary tip, and stars indicate the ciliary base.

To understand how ARL-3 and ARL-13 act antagonistically in ciliogenesis 10, we first examined the ciliary role of reported ARL interactors. UNC119B was previously shown to be an ARL3 interactor and regulate the proper ciliary targeting of lipid-modified proteins in mammalian cells^11^,^12^. Strikingly, we found GFP-tagged UNC-119 (UNC-119::GFP, the homolog of human UNC119B), localizes specifically to the middle segment of worm amphid and phasmid cilia, which completely recapitulates the localization pattern of GFP-tagged ARL-13 (ARL-13::GFP, Fig. 1a,b, Supplementary Fig. 1a,c)^10^. The N-terminal 86 a.a. but not the C-terminal Phosphodiesterase (PDE)-delta domain of UNC-119 accounts for its ciliary targeting (Supplementary Fig. 1a,d). In *C. elegans* amphid and phasmid cilia, the middle segment (or the doublet segment) is likely analogous to the recently identified but poorly understood mammalian Inversin (InV) compartment proximal to the transition zone^17^,^18^,^19^,^20^,^21^,^22^. Several cyclic nucleotide-gated (CNG) cation channel subunits were found exclusively in this InV-like compartment in worm cilia further supports this compartment is a functionally distinct domain^23^,^24^.

## UNC-119, ARL-3 and ARL-13 show mutual interactions with each other both *in vitro* and *in vivo*

Inspired by its distinct ciliary localization (Fig. 1a,b), we asked whether UNC-119 associates with ARL-13. Although not an InV-like compartment exclusive protein, ARL-3 was also included since mammalian ARL3 interacts with UNC119^25^. Intriguingly, ARL-3, ARL-13, and UNC-119 show mutual interactions with each other in GST pulldown (Fig. 1c,d). We mapped down the binding region and found that ARL-3 and UNC-119 associate with opposite sides of ARL-13. Specifically, ARL-13 interacts with ARL-3 through its N-terminus (a.a. 1–250 including both GTPase domain and coiled-coil domain), but binds with UNC-119 through its C-terminus (a.a.176–367 including both coiled-coil domain and proline rich domain (Fig. 1e–g). To confirm the associations happen *in vivo*, we performed bimolecular fluorescence complementation (BiFC) assay, which was developed for visualizing *in vivo* protein-protein interaction^26^. Compared to negative controls, strong fluorescence complementation was observed specifically in the InV-like compartment among all protein pairs for ARL-3/ARL-13/UNC-119, indicating *in vivo* mutual associations (Fig. 1h, Supplementary Fig. 1e).

We further found the mutual interactions among ARL-3-ARL-13-UNC-119 module do not require the palmitoylation^10^,^27^ or SUMOylation (SUMO: Small Ubiquitin-like Modifier)^9^ modification of ARL-13, or the activation of ARL-3 or ARL-13 (Supplementary Fig. 2a,b). This discovery is partly unexpected for UNC-119, since its mammalian homolog selectively associates with activated ARL3^25^. We confirmed that the BiFC signal of ARL-3(DN)-UNC-119 pair is not facilitated by endogenous proteins by confirming that ARL-3(DN)-UNC-119 still form strong fluorescence complementation in *unc-119, arl-3* double mutants (data not shown). This suggests that UNC-119 may not evolve as the effector of ARL-3 in lower ciliated organisms.

## ARL-13 is a key player in assembling the small GTPase module

**Figure 2:**
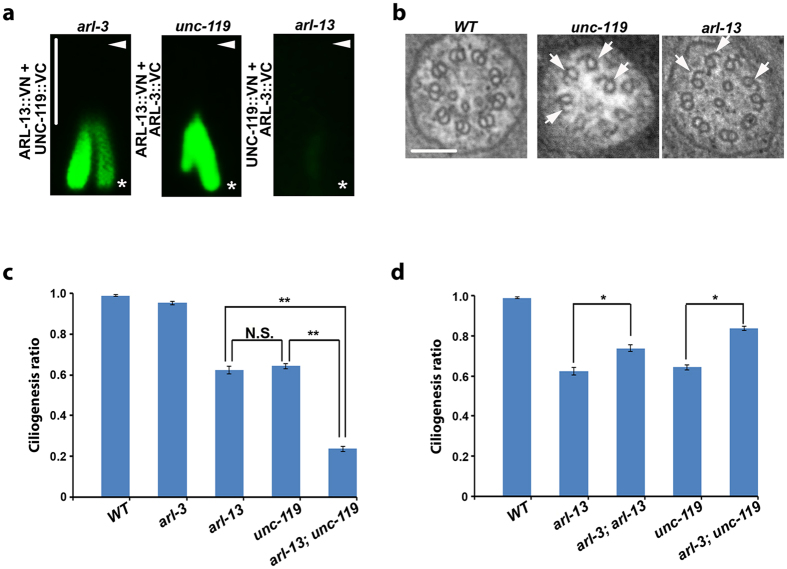
*unc-119, arl-13, and arl-3 coordinate ciliogenesis in C. elegans*. (**a**) Depletion of ARL-13, but not ARL-3 or UNC-119, disrupts the association between the other two components of UNC-119-ARL-13-ARL-3 functional module in cilia. VN: Venus N-terminus. VC: Venus C-terminus. All BiFC tags were fused to the carboxy-terminus of proteins. Arrowheads point to the ciliary tip. Stars point to the ciliary base. Scale bar: 5 μm. (**b**) Similar to *arl-13* mutants, *unc-119* cilia exhibit numerous B-tubule seam breaks. More than eight animals were sectioned for each genotype. Arrows point to the B-tubule seam breaks. (**c**) *unc-119* shows similar ciliogenesis defect as *arl-13. unc-119; arl-13* double mutants show synergic ciliogenesis defect. (**d**) *arl-3* depletion partially suppresses ciliogenesis defect in *arl-13* or *unc-119* single mutants. At least 100 animals (>400 cilia) were scored per genotype. * p < 0.01. ** p < 0.001. N.S. Not statistically significant.

To understand how the ARL-3-ARL-13-UNC-119 protein module assembles, we examined the ciliary entry of individual proteins as well as protein-protein associations in different mutants. Except for that mild mislocalization of ARL-13 was observed along the dendrite in *unc-119* mutants, depletion of one protein in ARL-3-ARL-13-UNC-119 module does not affect the ciliary entry of the other two components (Supplementary Fig. 2c,d). Remarkably, in BiFC assays, depletion of ARL-13 can totally disrupt ARL-3-UNC-119 association, whereas depletion of ARL-3 or UNC-119 does not affect the association between the other two proteins in cilia (Fig. 2a). Together with the fact that ARL-13 directly binds with ARL-3 and UNC-119 via its different terminus (Fig. 1e–g), we thus propose ARL-13 is crucial in assembling the small GTPase module.

## unc-119, arl-13, and arl-3 coordinate ciliogenesis in *C. elegans*

Transmission electron microscopy (TEM) analyses show that *unc-119* mutant cilia possess B-tubule seam breaks in microtubule doublets (Fig. 2b), which recapitulates the structural defect in either *arl-13* mutant worms or *Arl13b*^−/−^ mice^10^,^15^. Consistently, *unc-119* mutants show defective ciliogenesis similar to that of *arl-13* (Supplementary Fig. 1b). Since *arl-3* null show normal ciliogenesis and normal axonemal doublets (Fig. 2c, Supplementary Fig. 2e)^10^, we concluded that UNC-119 does not act as the effector of ARL-3 in C. elegans, at least during ciliogenesis. Further genetic analyses showed that *arl-13; unc-119* double mutants exhibit synthetic ciliogenesis defect, suggesting ARL-13 and UNC-119 genetically interact to regulate ciliogenesis (Fig. 2c). Similar to the observation that depletion of ARL-3 can rescue ciliogenesis defect of *arl-13*^10^, *arl-3; unc-119* double mutants also show restored ciliogenesis (Fig. 2d). These observations reveal an interesting model that the small GTPase module we identified here contains two positive regulators (ARL-13 and UNC-119) and one negative regulator (ARL-3) for cilia formation. However, it is worth to point out that the ciliogenesis defect observed in *arl-3* or *unc-119* mutant worm can only be partially rescued by ARL-3 deletion (Fig. 2d), which suggests that ARL-13 and UNC-119 probably have distinct functions in ciliogenesis that are independent of ARL-3.

## ARL-13 is a GEF for ARL-3

**Figure 3:**
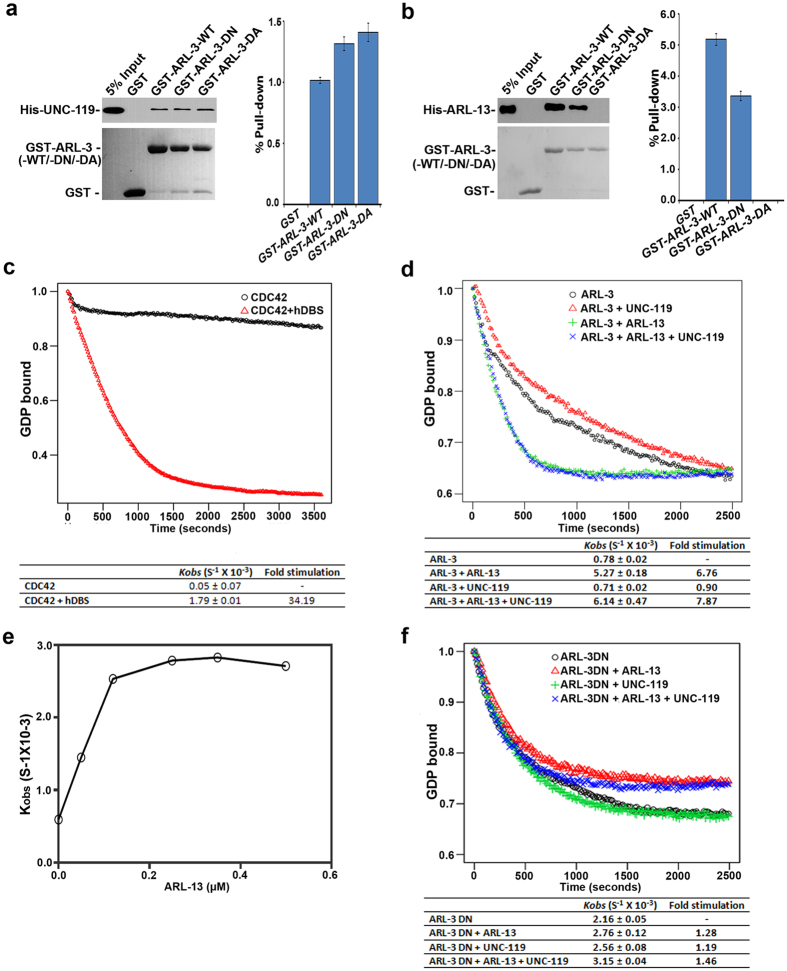
ARL-13 functions as a GEF for ARL-3. (**a**,**b**) GST-pull down assay shows that ARL-13 can only interact with dominant negative (DN, T31N) variant but not dominant active (DA, Q72L) variant of ARL-3. The binding affinity between UNC-119 and ARL-3 is not GTPases activity dependent. The input and pull-down samples were analyzed with immunoblotting *via* anti-His antibody (upper panel) and Ponceau S staining was used to indicate the GST proteins (lower panel). Quantifications of the pull down efficiency of different proteins were shown in bar graphs. The protein band intensities of individual pull-down sample were compared to the corresponding input sample via Image J to calculate percent of pull-down. (**c**) hDBS accelerates mantGDP release from CDC42 over 34 times. (**d**) ARL-3 shows a high intrinsic GDP release rate. ARL-13 accelerates the mantGDP release of ARL-3. Addition of UNC-119 does not affect the GDP release of ARL-3. (**d**) Hyperbolic dependence of the observed rate constants for mantGDP release of ARL-3 on various ARL-13 concentrations. The rate constants (*Kobs*) are plotted against ARL-13 concentrations. Individual *Kobs* values are shown in Supplemental Fig. 4a. (**f**) Replacing ARL-3 with ARL-3 DN totally abolishes the ARL-13-promoted GDP release. All GEF assays were normalized with the initial fluorescence reading. All experiments were repeated at least three times, and representative graphs were shown here.

Consistent with our BiFC results (Supplementary Fig. 2b), UNC-119 interacts with both dominant active (DA, Q72L) and dominant negative (DN, T31N) ARL-3 variants with comparable intensity in GST pulldown (Fig. 3a), confirming again that UNC-119 is not an effector of ARL-3 in worms. Intriguingly, ARL-13 can only interact with ARL-3 DN but not ARL-3 DA variant, suggesting a novel mechanism that one GTPase ARL-13 may act as a GEF for another GTPase ARL-3 (Fig. 3b).

We then performed *in vitro* GEF assay with fluorescent Methylanthraniloyl-labeled GDP (Mant-GDP). Recombinant proteins were expressed in *E. coli* and purified by affinity columns (Supplementary Fig. 3a). In contrast to conventional GTPase CDC42, GDP-preloaded GST-ARL-3 shows a very high intrinsic GDP release rate (Fig. 3c,d). WT CDC42 releases GDP at a 15-fold slower rate (*Kobs* = 0.05  ×  10^−3^•s^−1^) than ARL-3 (*Kobs* = 0.78 × 10^−3^•s^−1^). To confirm that GST tag does not cause the unusual high intrinsic GDP release rate, we repeated the experiment with His-tagged ARL-3 and observed similar results (Supplementary Fig. 3b). These data suggest that ARL-3 is an atypical GTPase with a high intrinsic GDP release rate. Nevertheless, addition of ARL-13 to GDP-preloaded ARL-3 in a 1:1 molar ratio can increase the GDP release ~7-fold over controls, indicating that GTPase ARL-13 does function as the GEF for another GTPase ARL-3 (Fig. 3d, Supplementary Fig. 3b). We then tested whether the GDP dissociation of ARL-3 accelerated by ARL-13 is dependent on the ARL-13 concentration. We found that ARL-13 is very efficient in promoting GDP release from ARL-3. ARL-13 can stimulate ARL-3 GDP release ~2 fold with even one-tenth molar of ARL-3. And its activity reaches plateau with one-fourth molar of ARL-3 (Fig. 3e, Supplementary Fig. 4a). As a control we showed that ARL-3•GTP does not prompt the mantGppNHp release of ARL-13 (Supplementary Fig. 4b). Replacing ARL-3 with ARL-3 DN totally abolishes ARL-13-promoted GDP release, suggesting that the increased GDP release observed with ARL-3-ARL-13 sample is mediated by ARL-3 but not ARL-13 (Fig. 3f). Since only the N-terminus of ARL-13 interacts with ARL-3, we further tested which fragment of ARL-13 is responsible for its GEF activity. As expected, only the first 1–250 a.a of ARL-13, which interacts with ARL-3, is capable of accelerating the mantGDP release from ARL-3 (Supplementary Fig. 4c,d). Those results together confirm that GTPase ARL-13 does function as the GEF for another small GTPase ARL-3.

## UNC-119 stabilizes the GTP-binding of ARL-3 activated by ARL-13

**Figure 4:**
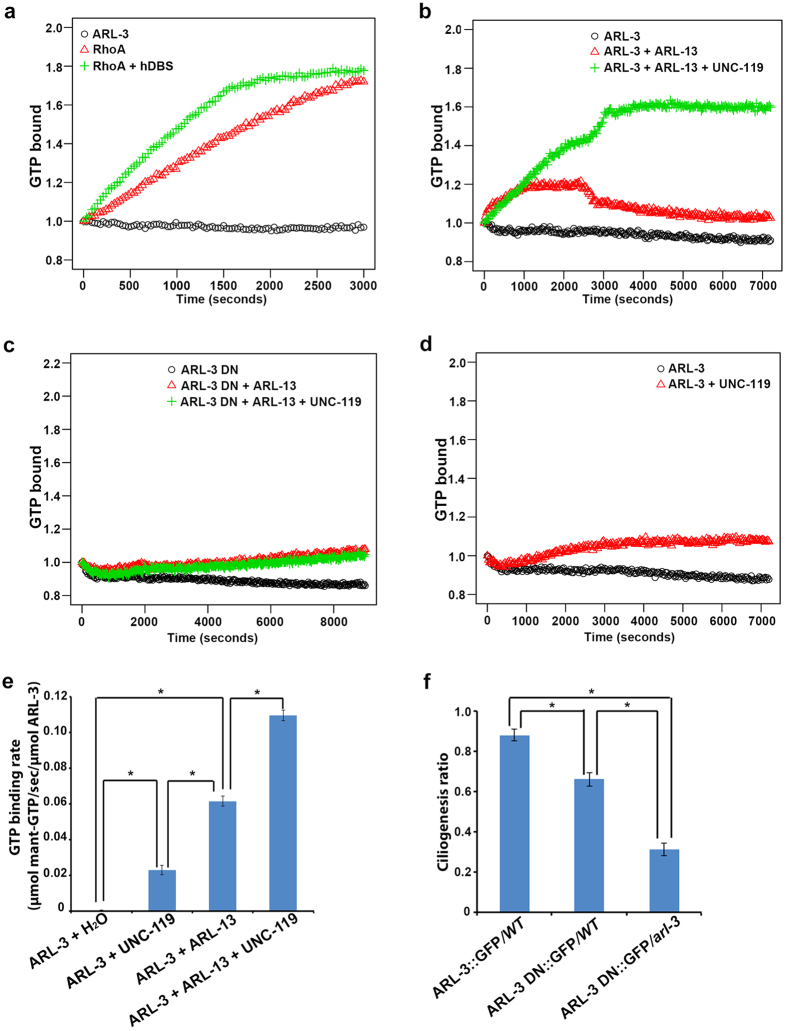
UNC-119 stabilizes the GTP binding of ARL-3. (**a**) Compare to RhoA, ARL-3 shows little intrinsic GTP binding. (**b**) ARL-13 alone can promote the GTP binding of ARL-3, but this binding is not stable. UNC-119 is needed for stabilizing the binding between ARL-3 and GTP. (**c**) Neither ARL-13 or ARL-13 + UNC119 can promote GTP binding of ARL-3 DN, suggesting that ARL-3, but not ARL-13, is responsible for GTP-binding activity. (**d**) Without ARL-13 functioning as a GEF, UNC-119 alone can promote GTP binding at a much slower rate. (**e**) GTP binding rates were calculated (see methods). (**f**) Ciliogenesis analyses show that excessive amount of ARL-3 DN lead to ciliogenesis defect, with more severe phenotype observed in *arl-3* mutants than in *Wt* background. At least 40 animals (>160 cilia) were scored per genotype. All GTP binding assay were normalized with the initial fluorescence reading. All experiments were repeated at least three times, and representative graphs were shown here. *p < 0.001.

Adding UNC-119 alone or together with ARL-13 does not show or enhance GEF activity for ARL-3 (Fig. 3d, Supplementary Figs 3b and 4d). GEF assay can use either Mant-GDP to monitor GDP release rate or Mant-GTP to monitor GTP binding rate. In contrast to RhoA as a positive control, ARL-3 shows negligible intrinsic GTP-binding (Fig. 4a). Surprisingly, distinct from the positive controls, the GTP binding of ARL-3 with the presence of GEF ARL-13 only increases temporarily and then drops slowly (Fig. 4b). We did not detect unusual GTP hydrolysis and thus excludes the possibility that the atypical GTP loading of ARL-3-ARL-13 group is due to high intrinsic GTP hydrolysis by ARL-3 or ARL-13 (Supplementary Fig. 5a,b). Intriguingly, adding UNC-119 together with ARL-13 stabilize the GTP binding of ARL-3 (Fig. 4b). GTP-binding rate for ARL-3 was approximately 100% faster with the presence of both ARL-13 and UNC-119 compared to that with ARL-13 alone (Fig. 4e). As expected, replacing WT ARL-3 with ARL-3 DN totally abolishes the GTP-binding (Fig. 4c). Surprisingly, UNC-119 alone can also slowly promote the GTP-binding of ARL-3 at a ~3-fold slower rate than ARL-13 (Fig. 4d,e). Since UNC-119 is not the GEF for ARL-3 (Fig. 3d), we reasoned that UNC-119 does not affect the nucleotide exchange of ARL-3, but rather stabilizes ARL-3-GTP by spatial and conformational regulation. Taken together, we conclude that ARL-3 is a unique small GTPase with high intrinsic GDP release but low GTP binding. This is distinct from other atypical small GTPases (RhoU and RhoV, *etc*) that possessing high intrinsic rate for both GDP release and GTP binding^28^,^29^. Also, the coordination between ARL-13 and UNC-119 to activate and maintain the GTP-binding of ARL-3 is consistent with our genetic observations that *unc-119* acts synergistically with *arl-13*, but antagonistically with *arl-3*, in regulating ciliogenesis.

We previously hypothesized that depletion of activated ARL-3 in ARL-13-deficient cilia might be beneficial for ciliogenesis and explain improved ciliogenesis in *arl-3; arl-13* double mutants^10^. Based on our current data, it is more likely that defective ciliogenesis in *arl-13* or *unc-119* is caused by excess inactivated ARL-3 instead. We reason that the dosage of inactivated ARL-3 in cilia should be correlated to the severity of ciliogenesis defect. Indeed, overexpressing ARL-3 DN variant, which localizes normally to cilia (Supplementary Fig. 5c), does cause more severe ciliogenesis defect in *arl-3* mutants than in *Wt* background (Fig. 4f). ARL-3 itself is not required for ciliogenesis since *arl-3* mutants possess normal cilia formation. We thus conclude that excess inactivated ARL-3 may bind and titrate out key players involved in ciliogenesis and thus compromises ciliogenesis indirectly. Many small GTPases work coordinately in the context of cilia. Unfortunately, we know little about how these small GTPases are activated/deactivated in the context of cilia. Excess inactivated GTPases may titrate out their GEF or other unknown proteins. For example, ARF1-GDP specifically recognizes members of the p24 family of transmembrane proteins to regulate the formation of transport vesicles^30^,^31^. It is possible that excess inactivated ARL-3 may bind ARL-13 or other key players and make them unavailable for ciliogenesis.

Here we identify novel roles for Joubert syndrome protein ARL-13 in acting as a GEF of ARL-3, and for UNC-119 in stabilizing ARL-3-GTP binding. One recent study also suggests that small GTPase IFT27/RABL4, although not as a GEF, can mildly promote the GDP release of ARL6^32^. Thus, an interesting question is that whether using another small GTPase as the GEF or, part of the GEF is a unique biochemical feature for ARL GTPases.

Ciliary protein modules play indispensable roles in regulating many stereotyped steps that happened sequentially during ciliogenesis. For example, the anchoring of the basal body requires protein modules on transition fibers (TFs)^33^,^34^. After ciliogenesis initiation, NPHP and MKS protein modules coordinate the formation and function of the transition zone (TZ)^35^,^36^, and then IFT complexes build and maintain cilia^36^,^37^. The fact that the distinct functional domain that ARL-3-ARL-13-UNC-119 defined is near the InV-like compartment and proximal to the ciliary base is in good agreement with their potential roles in regulating IFT and/or the ciliary entry^9^,^10^,^11^,^13^,^18^,^27^,^38^,^39^,^40^. Dissecting the composition and function of ciliary protein modules could be a fast-lane approach to understand how cilia form and function as well as the pathogenesis of ciliopathies. Moreover, the fact that ARL3 and ARL13B are enzymatic proteins also promises the potential to be used as therapeutic targets for future clinical development.

## Materials and Methods

### Strains, Constructs and Maintenance

Nematodes were raised using standard conditions. N2 worms represented wild-type animals in all assays described in this study. To ensure the expression level of studied proteins are comparable between wide-type and mutants, transgenic strain were generated in wild-type N2 worms by injecting low concentration of plasmids. All tagged protein used in *C. elegans* experiments have tags fused to C-terminus. All strains used in this study are listed in supplementary Table S1.

### Microscopy

The live worms were mounted on 5% agar pads by anaesthetization in 10mM levamisole (diluted in M9 buffer) and taken images under a Plan Apochromat 60X, 1.49 NA oil objective (Nikon) with an imaging microscope (TE 2000-U, Nikon).

### Ciliogenesis Assay

In *C. elegans*, amphid and phasmid cilia can take up lipophilic fluorescent dye from outside environment. Worm mutants with abnormal ciliogenesis fail to take up dye and are thus called dye-filling defective. Hermaphrodites were washed of plates with M9, and then incubated in 10 μg/mL DiI (2 mg/mL in dimethyl formamide, diluted 1:200 in M9; Invitrogen) for 2 h at room temperature in the dark. After incubation, the animals were washed at least three times with M9, and then placed on a plate for a further 10–30 minutes to recover. Animals were anesthetized and then immediately scored using a fluorescence microscope for dye filling.

### Visualization of *in vivo* protein-protein association using BiFC assay

The Venus-based Bimolecular fluorescence complementation (BiFC) analysis was performed to detect the protein associations in cilia from both worms and cells. For BiFC assay in worms, BiFC vectors pCE_BiFC_VN173 and pCE_BiFC_VC155 vectors were used. Worm proteins were cloned in BiFC vectors, respectively. BiFC pairs were microinjected into N2 worms to generate transgenic animals. To examine the BiFC signals relative to transition zone, plasmids in the combinations were co-injected along with the TZ marker MKS-5::mCherry and co-injection marker pRF4 (rol-6(su1006)) into wide-type worms (20 ng/μl for each BiFC plasmid and MKS-5 vector, 30 ng/μl pRF4). Once a stable line was obtained, the same transgene was crossed into various mutants. Fluorescent signals were visualized by using the YFP filter.

### GST-Pull down assay

The full-length and/or truncated cDNA of ARL-3 (including wide-type-, DN- and DA-forms), ARL-13, and UNC-119 were cloned into pGEX-4T1 and pET28a vectors, respectively. The N-terminal GST- and His-tagged proteins were expressed in *E. coli* BL21cells and purified using His resin or glutathione sepharose. The purified proteins were used for the assay and GST alone as a negative control. Briefly, the His-tagged protein was incubated with GST, GST-tagged proteins immobilized on glutathione sepharose in the binding buffer (25 mM Tris-HCl, pH 7.4, 150 mM NaCl, 0.5% Triton X-100, 1 mM DTT, 10% glycerol, and protease inhibitors) for 4 hours at 4 °C. The beads were then washed six times with binding buffer and analyzed by western blot. Anti-His antibody was used for the immunoblotting and Ponceau S staining was used to confirm the protein amount.

### Guanine nucleotide exchange assays

Preloading CDC42, ARL-3, or ARL-3DN (T31N) with Mant-GDP (Life technologies) was done by using 5 mM EDTA, same molar Mant-GDP in binding buffer (100 mM Tris-HCL, pH 7.5, 100 mM NaCl, and 1 mM DTT) incubating 30 min at 30 °C, then stopped with 20 mM MgCl_2_. Excess nucleotide was removed by desalting columns, 7 K MWCO (Thermo Scientific). The protein concentrations were measured by Bradford assay (BioRed). For nucleotide exchange assay, 0.5 μM mantGDP-bound proteins were incubated with either 0.5 μM GEF or buffer in exchange buffer (20 mM HEPES, pH 7.5, 100 mM NaCl, 1 mM DTT, 20 mM MgCl_2_) and the reaction was started with 20 times excess unlabeled GTP (Life technologies) at 25 °C. Time-dependent fluorescence change was recorded at λex = 366 nm, λem = 450 nm using Synergy H1 (Biotek). The rate constants (*Kobs*) of GDP release were determined by fitting the data as one phase exponential decay using Prism 6.

*In vitro* GTP binding assay was carried out by using RhoGEF Mant-GTP exchange assay Kit (Cytoskeleton, Inc.) according to the manufacturer’s instructions. Briefly, spectroscopic analysis of Mant-GTP incorporation into 4 μM purified ARL-3, or RhoA, or ARL-3DN (T31N), with or without 1.3 μM GEF was carried out using Synergy H1 at 25 °C (λex = 360 nm, λem = 440 nm). Exchange rates were calculated as manufacturer’s instructions (Exchange rate = Vmax (AFU/sec)/0.75 × Basal mant-GTP AFU × N). Vmax were determined by Gene5 software accompanied with Synergy H1. The N value for ARL-3 is not available yet. We used the N value (N = 2) of CDC42 in all calculations.

### GAP assay

GTP hydrolysis assays were carried out by using RhoGAP Kit (Cytoskeleton, Inc.) according to the manufacturer’s instructions. Reactions were incubated at room temperature or 37 °C for 60 min. Phosphate generated by hydrolysis of GTP was measured by the addition of CytoPhos™ reagent and reading of absorbance at 650 nm by Synergy H1.

### Statistical analysis

We assume the number of dye-filling cilia follows a binomial distribution for each worm strain with a probability parameter representing the probability being dye-filling cilia. Then we test the null hypothesis that the probability parameters are the same for each pair of worm strains of interest. The total cilia counts and dye-filling positive cilia counts for different worm strains were fitted in a logistic model and multiple comparisons for specified pair of worm strains were performed. Bonferroni adjustment was used for these multiple comparisons. We also estimated the 95% confidence interval for the probability parameter for each strain. All tests were performed in R using the R package car and effects (R Core Team (2014). R: A language and environment for statistical computing. R Foundation for Statistical Computing, Vienna, Austria. URL http://www.R-project.org/).

## Additional Information

**How to cite this article**: Zhang, Q. *et al*. GTP-binding of ARL-3 is activated by ARL-13 as a GEF and stabilized by UNC-119. *Sci. Rep.* **6**, 24534; doi: 10.1038/srep24534 (2016).

## Supplementary Material

Supplementary Information
