# Supplementary material for: GTP-binding of ARL-3 is activated by ARL-13 as a GEF and stabilized by UNC-119
Source: Sci Rep. 2016 Apr 22;6:24534. doi: 10.1038/srep24534 (PMC4840320; doi:10.1038/srep24534)
Supplement: Supplementary Information [file srep24534-s1.pdf]

Title: GTP-binding of ARL-3 is activated by ARL-13 as a GEF and stabilized by UNC-119.

Qing Zhang[1,3#], Yan Li[1,3#], Yuxia Zhang[1,3], Vicente Torres[1,2], Peter Harris[1,2,3], Kun Ling[3], Jinghua Hu[1,2,3,] *

Supplementary Materials:

Supplementary Figure legends

Figure S1. UNC-119 localizes to the InV-like compartment of cilia. (**a**)**.** UNC-119 is highly conserved across ciliated species. ***Ce***, *Caenorhabditis elegans*; ***Cr**, Chlamydomonas reinhardtii*; ***Dr***: *Danio rerio*r; ***Mm***, *Mus musculus*; ***Hs***, *Homo sapiens*. (**b**)**.** *unc-119 (ed3)* mutants are dye-filling defective in phasmid cilia. The wild-type copy of GFP-tagged UNC-119 used in localization studies can fully rescue ciliogenesis defect of *unc-119* mutants. *: $p < 0.01$. (**c**)**.** GFP-tagged UNC-119 only localizes along the middle segment. mCherry-tagged β-tubulin TBB-4 was used to label the whole cilium. (**d**)**.** The N-terminal sequence but not the PDE-delta domain is required for UNC-119 ciliary targeting. (e) Negative controls for BiFC studies. UNC-119, ARL-13, and ARL-3 do not show fluorescence complementation with IFT components (CHE-11 and IFT-20) in BiFC studies. Scale bar: 5 μm. Arrowheads indicate the ciliary tip, and stars indicate the ciliary base.

Figure S2. Characterization of UNC-119-ARL-13-ARL-3 association. (**a**)**.** The Palmitoylation or SUMOylation modification of ARL-13 is not required for its association with UNC-119 or ARL-3 in BiFC study. (**b**)**.** Dominant negative ARL-3 or Dominant negative ARL-13 still show normal mutual interactions with other components in the UNC-119-ARL-13-ARL-3 module in BiFC study**.** (**c**)**.** Deletion of UNC-119, or ARL-13, or ARL-3 does not affect the ciliary entry of other proteins in the UNC-119-ARL-13-ARL-3 module. mCherry-tagged MKS-5 was used to label the transition zone at the ciliary base. Arrows point to the abnormal bulge formed in residual cilia in *arl-13* mutants. (**d**)**.** ARL-13 exclusively localizes to the middle segment of cilia in wide-type worms and mislocalizes to the dendrite and the cell body in *unc-119* mutants. **E.** *arl-3* mutants possess normal axonemal doublets. Scale bars: (**a-d**), 5 μm; **E**, 100 nm. Arrowheads indicate the ciliary tip, and stars indicate the ciliary base.

Figure S3. ARL-13 promotes GDP release from ARL-3. (**a**)**.** All purified proteins used in this manuscript were showed here by SDS-PAGE and stained by Coomassie blue. 5μg protein was loaded in each lane. BSA served as indication for the protein amount. (**b**)**.** ARL-13 but not UNC-119 accelerates GDP release from His-tagged ARL-3.

Figure S4. The N terminal but not the C terminal domain of ARL-13 can accelerates GDP release from GST-ARL-3. (a). GEF activity of the indicated concentrations of ARL-13 for 0.05 μM ARL-3·mantGDP. *Kobs* ($s^{-1}\times10^{-3}$) and fold change are shown in the table below the graph. (**b**). ARL-3·GTP cannot promote nucleotide dissociation of ARL-

13·mantGppNHp. (**c**). The N-terminal domain of ARL-13 containing the GTPase domain (1-176aa) and the coil-coil domain (177-250aa) is able to accelerate the GDP release from ARL-3·mantGDP. (**d**). Adding UNC-119 together with FL or truncated ARL-13 does not change the nucleotide exchange rate of ARL-3.

Figure S5. ARL-3 and ARL-13 show no intrinsic GTP hydrolysis activity. No GTP hydrolysis was detected when mixing Mant-GTP with either ARL-3 or ARL-3 +ARL-13 at room temperature (**a**)or 37°C (**b**)for 60 min. RhoA and RhoA GAP (P50) served as positive control. (**c**)**.** GFP-tagged ARL-3(T31N) normally localizes in cilia. mCherry-tagged MKS-5 was used to label the transition zone at the ciliary base. Scale bars: 5 µm; The arrowhead indicates the ciliary tip, and the star indicates the ciliary base.

Supplementary Fig. 1

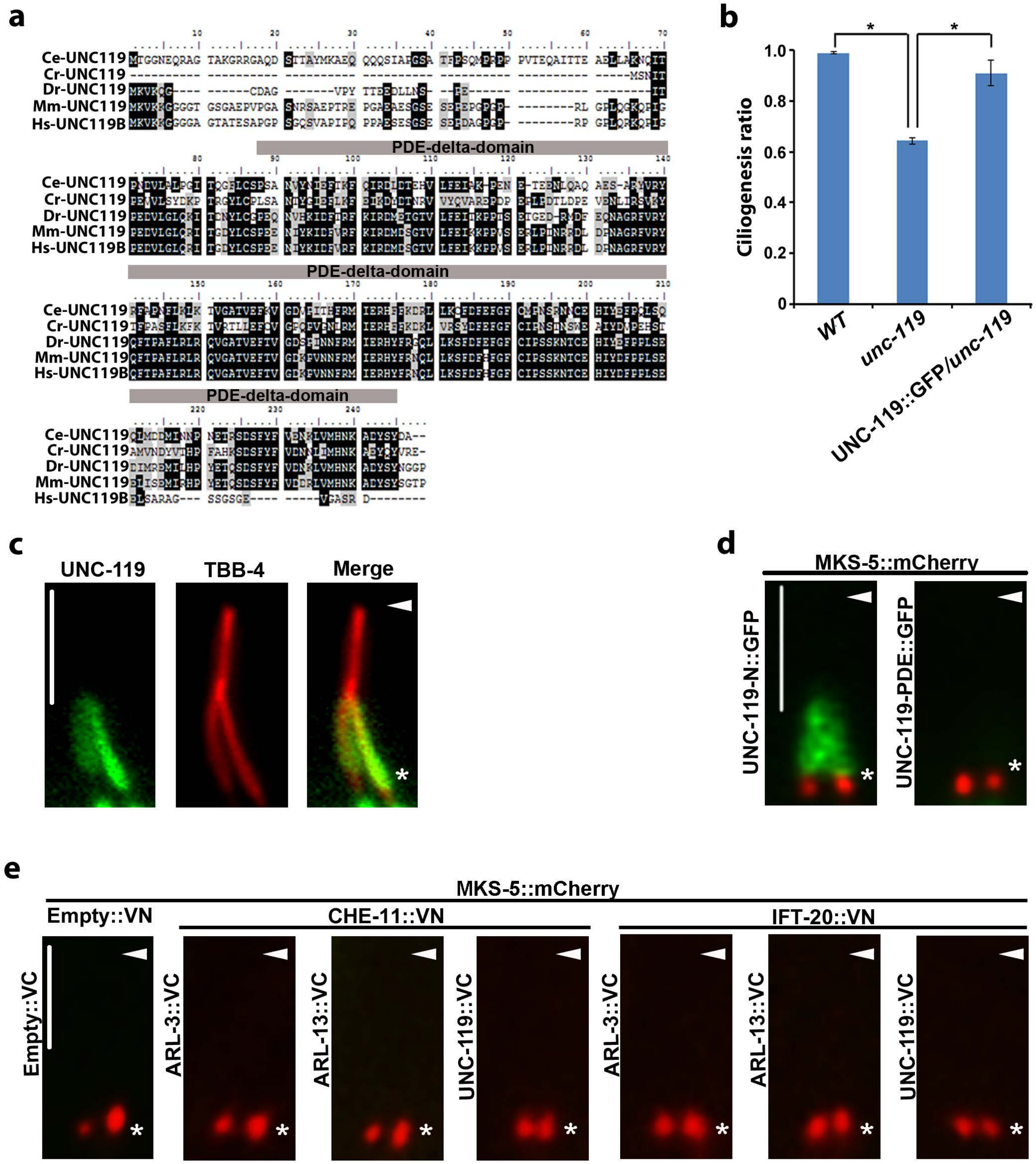

Supplementary Fig. 2

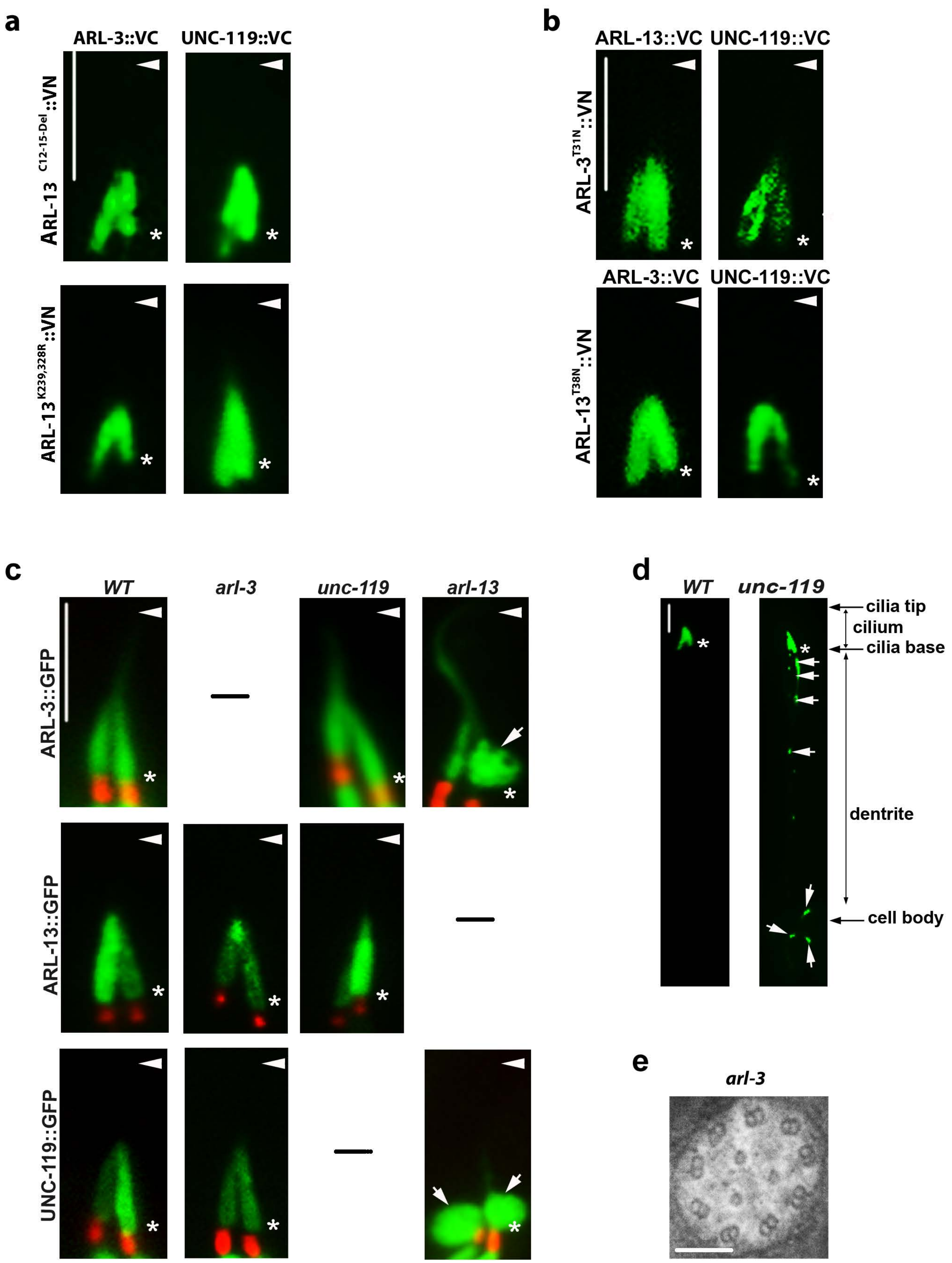

Supplementary Fig. 3

a

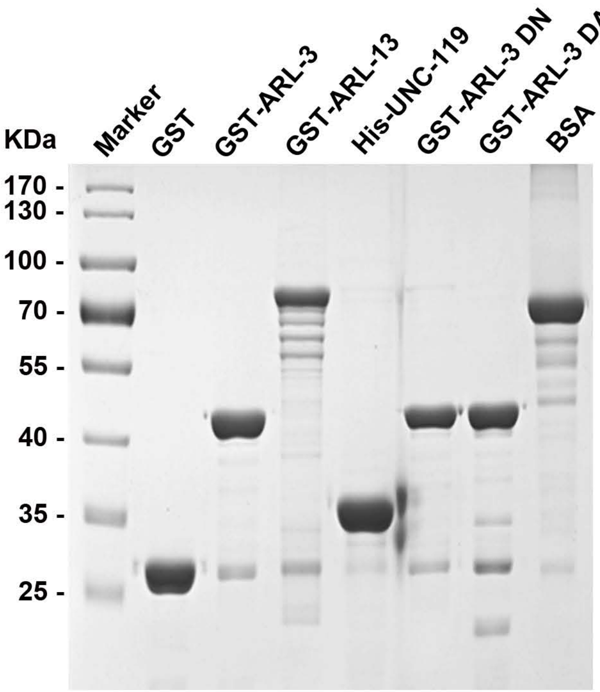

b

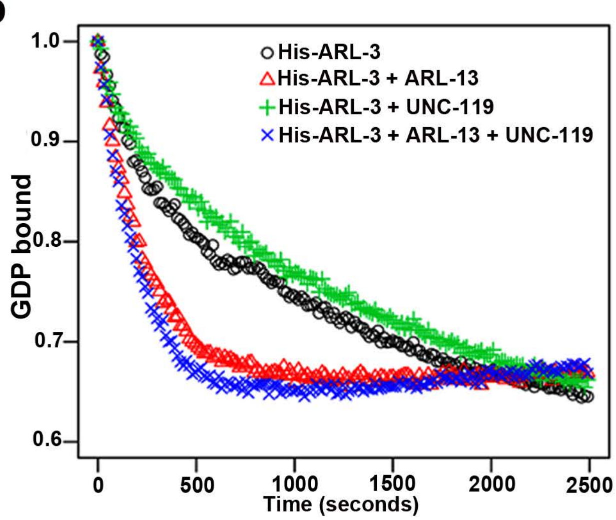

	Kobs (S^{-1} X 10^{-3})	Fold stimulation
His-ARL-3	1.07 ± 0.03	-
His-ARL-3 + ARL-13	4.74 ± 1.13	4.45
His-ARL-3 + UNC-119	0.83 ± 0.04	0.78
His-ARL-3 + ARL-13 + UNC-119	7.66 ± 0.43	7.19

Supplementary Fig. 4

a

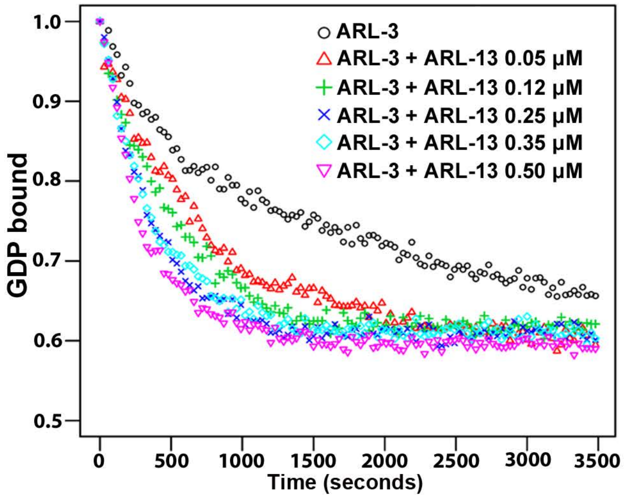

mantGDP-GST-ARL-3		
	Kobs (S^{-1}×10^{-3})	Fold stimulation
None	0.59 ± 0.03	-
ARL-13 0.05μM	1.45 ± 0.06	2.44
ARL-13 0.12μM	2.53 ± 0.36	4.26
ARL-13 0.25μM	2.78 ± 0.16	4.69
ARL-13 0.35μM	2.83 ± 0.08	4.76
ARL-13 0. 5μM	2.71 ± 0.06	4.57

b

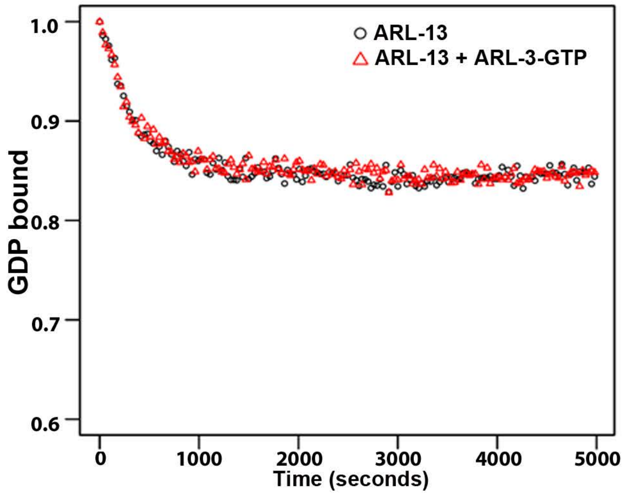

mantGppNHp-GST-ARL-13		
	Kobs (S^{-1}×10^{-3})	Fold stimulation
None	2.87 ± 0.13	-
ARL-3-GTP	2.66 ± 0.22	0.93

c

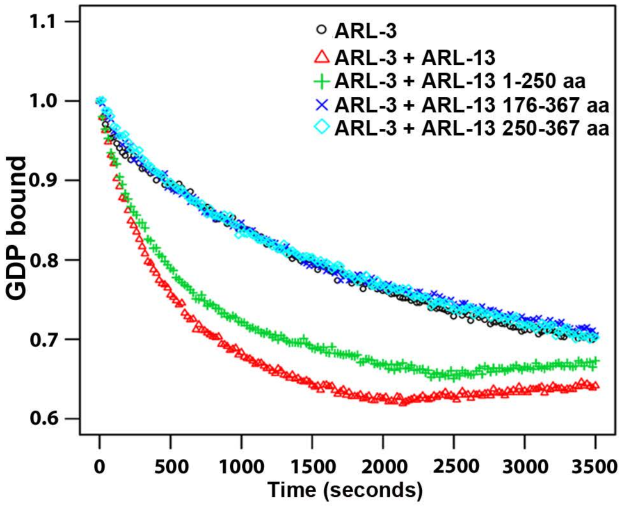

mantGDP-GST-ARL-3		
	Kobs (S^{-1}×10^{-3})	Fold stimulation
None	0.50 ± 0.02	-
ARL-13	1.79 ± 0.03	3.57
ARL-13 1-250aa	1.38 ± 0.04	2.74
ARL-13 176-367aa	0.51 ± 0.03	1.01
ARL-13 250-367aa	0.58 ± 0.04	1.16

d

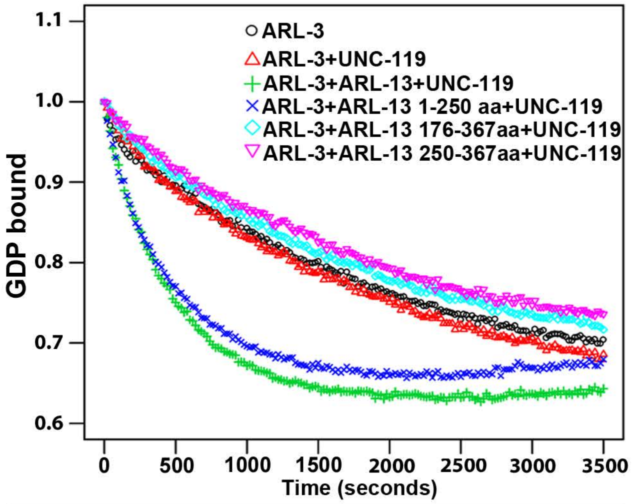

mantGDP-GST-ARL-3		
	Kobs (S^{-1}×10^{-3})	Fold stimulation
None	0.50 ± 0.02	-
ARL-3 + UNC-119	0.50 ± 0.01	0.98
ARL-13 + UNC-119	2.23 ± 0.10	4.44
ARL-13 1-250aa + UNC-119	2.06 ± 0.03	4.11
ARL-13 176-367aa + UNC-119	0.50 ± 0.03	1.00
ARL-13 250-367aa + UNC-119	0.50 ± 0.01	0.99

Supplementary Fig. 5

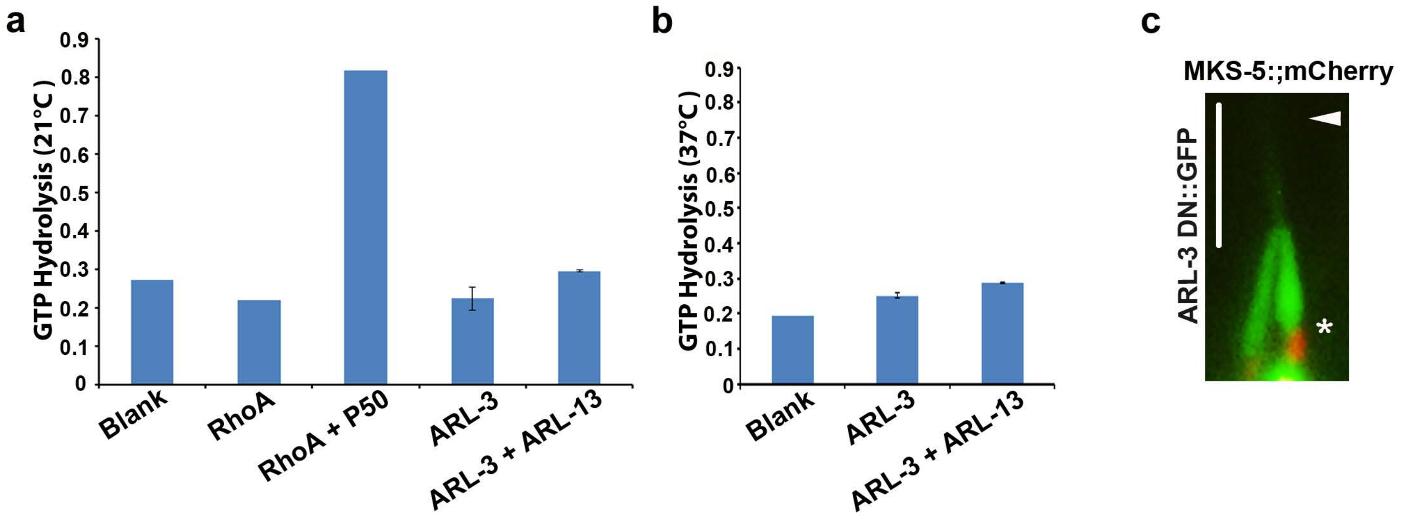

Table S1. Transgenic strains used in this study

Strain name	Genotype
ZP1863	*arl-3 (tm1703); arl-13 (gk513)*
ZP1865	*arl-3 (tm1703); arl-13 (gk513); unc-119 (ed3)*
ZP1866	*arl-13 (gk513); [Parl-13::UNC-119::GFP; Parl-13::TBB-4::mCherry; PR4]*
ZP1867	*arl-3 (tm1703); [UNC-119::VN; ARL-13::VC; PR4]*
ZP1870	*N2; [ARL-13T38N::VN; ARL-3::VC; PR4]*
ZP1871	*N2; [ARL-13T38N::VN; UNC-119::VC; PR4]*
ZP1886	*arl-3 (tm1703); unc-119 (ed3)*
ZP1887	*unc-119 (ed3); [Parl-13::ARL-13::GFP]*
ZP1889	*arl-13 (gk513); [ARL-3::VN; UNC-119::VC; RP4]*
ZP1892	*arl-13 (gk513); him8*
ZP1893	N_2 *[ARL-13::VN; ARL-3::VC; ccGFP]*
ZP1895	N_2 *[ARL-3::VN; UNC-119::VC; ccGFP]*
ZP1900	N_2 *[ARL-13::VN; UNC-119::VC; MKS-5::mCherry; ccGFP]*
ZP1901	N_2 *[ARL-13::VN; ARL-3::VC; MKS-5::mCherry; ccGFP]*
ZP1905	N_2 *[ARL-3::VN; UNC-119::VC; MKS-5::mCherry; ccGFP]*
ZP1910	*arl-3 (tm1703); OSM-6::GFP*
ZP1911	*unc-119 (ed3); OSM-6::GFP*
ZP1912	*arl-3 (tm1703); arl-13 (gk513); OSM-6::GFP*
ZP1913	*arl-3 (tm1703); unc-119 (ed3); OSM-6::GFP*
ZP1914	*unc-119 (ed3); OSM-6::GFP*
ZP1916	N_2 *[Parl-13::ARL-3::GFP; MKS-5::mCherry; PR4]*
ZP1917	*unc-119 (ed3) [Parl-13::ARL-3::GFP; MKS-5::mCherry; PR4]*
ZP1919	N_2 *[Parl-13::UNC-119::GFP; MKS-5::mCherry; PR4]*
ZP1920	*arl-3 (tm1703); [Parl-13::UNC-119::GFP; MKS-5::mCherry; PR4]*
ZP1921	*arl-13 (gk513); [Parl-13::UNC-119::GFP; MKS-5::mCherry; PR4]*
ZP1926	*unc-119 (ed3); [Parl::ARL-13::GFP]*
ZP1927	*arl-3 (tm1703); [Parl::ARL-13::GFP]*
ZP1953	*arl-13 (gk513); unc-119 (ed3)*
ZP1959	N_2 *[Parl-13::ARL-13::GFP; MKS-5::mCherry; PR4]*
